# Associations of local white matter geometry with network efficiency, macrostructural abnormalities, and clinical severity in behavioural variant frontotemporal dementia

**DOI:** 10.1093/braincomms/fcag226

**Published:** 2026-06-16

**Authors:** Qinyao Sun, Yu Zhang, Xin Jin, Jian Cheng, Jianyu Li, Ting Qiu, Zhanbing Ren, Ke Li, Huixiong Zhang, For the Frontotemporal Lobar Degeneration Neuroimaging Initiative, Howard Rosen, Bradford C Dickerson, Kimoko Domoto-Reilly, David Knopman, Bradley F Boeve, Adam L Boxer, John Kornak, Bruce L Miller, William W Seeley, Maria-Luisa Gorno-Tempini, Scott McGinnis, Maria Luisa Mandelli, Kewei Chen, Lena Palaniyappan, Yifan Chen, B Blair Braden, Yuanchao Zhang

**Affiliations:** The Clinical Hospital of Chengdu Brain Science Institute, MOE Key Lab for Neuroinformation, University of Electronic Science and Technology of China, Chengdu 611731, P. R. China; School of Life Science and Technology, University of Electronic Science and Technology of China, Chengdu, Sichuan 610054, P. R. China; School of Psychology, Shanghai Jiao Tong University, Shanghai 200030, China; The Clinical Hospital of Chengdu Brain Science Institute, MOE Key Lab for Neuroinformation, University of Electronic Science and Technology of China, Chengdu 611731, P. R. China; School of Life Science and Technology, University of Electronic Science and Technology of China, Chengdu, Sichuan 610054, P. R. China; School of Computer Science and Engineering, Beihang University, Beijing 100191, China; The Clinical Hospital of Chengdu Brain Science Institute, MOE Key Lab for Neuroinformation, University of Electronic Science and Technology of China, Chengdu 611731, P. R. China; School of Life Science and Technology, University of Electronic Science and Technology of China, Chengdu, Sichuan 610054, P. R. China; Douglas Mental Health University Institute, McGill University, Montreal H4H 1R3, Canada; College of Physical Education, Shenzhen University, Shenzhen 518060, P. R. China; The Clinical Hospital of Chengdu Brain Science Institute, MOE Key Lab for Neuroinformation, University of Electronic Science and Technology of China, Chengdu 611731, P. R. China; School of Life Science and Technology, University of Electronic Science and Technology of China, Chengdu, Sichuan 610054, P. R. China; The Clinical Hospital of Chengdu Brain Science Institute, MOE Key Lab for Neuroinformation, University of Electronic Science and Technology of China, Chengdu 611731, P. R. China; School of Life Science and Technology, University of Electronic Science and Technology of China, Chengdu, Sichuan 610054, P. R. China; College of Health Solutions, Arizona State University, Tempe, AZ 85281, USA; Douglas Mental Health University Institute, McGill University, Montreal H4H 1R3, Canada; The Clinical Hospital of Chengdu Brain Science Institute, MOE Key Lab for Neuroinformation, University of Electronic Science and Technology of China, Chengdu 611731, P. R. China; School of Life Science and Technology, University of Electronic Science and Technology of China, Chengdu, Sichuan 610054, P. R. China; College of Health Solutions, Arizona State University, Tempe, AZ 85281, USA; The Clinical Hospital of Chengdu Brain Science Institute, MOE Key Lab for Neuroinformation, University of Electronic Science and Technology of China, Chengdu 611731, P. R. China; School of Life Science and Technology, University of Electronic Science and Technology of China, Chengdu, Sichuan 610054, P. R. China; College of Health Solutions, Arizona State University, Tempe, AZ 85281, USA

**Keywords:** behavioural variant frontotemporal dementia, director field analysis, microstructure, network efficiency, proteinopathy

## Abstract

Behavioural variant frontotemporal dementia (bvFTD), marked by profound changes in behaviour and personality, is the most common subtype of frontotemporal dementia, driven by neurodegeneration in frontotemporal regions. This neurodegeneration pattern is partially shaped by white matter abnormalities arising from the spread of protein aggregates along axonal pathways. While prior studies mainly focused on diffusion tensor imaging metrics such as fractional anisotropy and mean diffusivity, the alteration in local white matter geometry remains largely unexplored. Using a novel Director Field Analysis (DFA) method, 51 patients with bvFTD and 51 healthy controls were studied to examine alterations in the local geometry of white matter fibres in bvFTD, and their associations with macrostructural morphology, global network parameters, and clinical manifestations. Unlike the unidirectional decrease in fractional anisotropy and increase in mean diffusivity, we identified significant bidirectional alterations in white matter local geometry, characterized by increased geometric distortion in the forceps minor and dorsal cingulum and decreased distortion in widespread frontotemporal association tracts, including the inferior fronto-occipital fasciculus, superior longitudinal fasciculus, uncinate fasciculus, frontal aslant tract, and arcuate fasciculus. Patients with bvFTD also showed reduced cerebral white and grey matter volumes (both *P* < 0.0026), enlarged lateral ventricles and choroid plexus (both *P* < 0.0001), decreased global network efficiency (*P* = 0.0010), and increased local efficiency (*P* = 0.0014). Importantly, decreased white matter geometric distortion across affected tracts was strongly associated with greater clinical severity, as reflected by higher Clinical Dementia Rating scores (*r* = −0.68, *P* < 0.0001). Mediation analyses further demonstrated that white matter geometric distortion significantly mediated the effects of macrostructural atrophy and reduced global network efficiency on clinical severity. Furthermore, neuroimaging-transcriptional association analysis on the group differences in nodal efficiency of the white matter networks identified several biological processes/pathways critical for the formation and propagation of TAR-DNA-binding protein 43/microtubule-associated protein tau pathologies along axonal pathways, as well as processes related to cellular homeostasis and oligodendrocyte-related pathways that may exacerbate these proteinopathies. Our findings advance understanding of the neural bases of the functional impairments in bvFTD and suggest potential mechanistic pathways for developing novel treatment strategies.

## Teaser

Sun *et al*. report bidirectional alterations in white matter wiring geometry in behavioural variant frontotemporal dementia. These geometric changes link macrostructural brain abnormalities and large-scale network disruption to the severity of clinical symptoms.

## Graphical Abstract

**Graphical Abstract:**
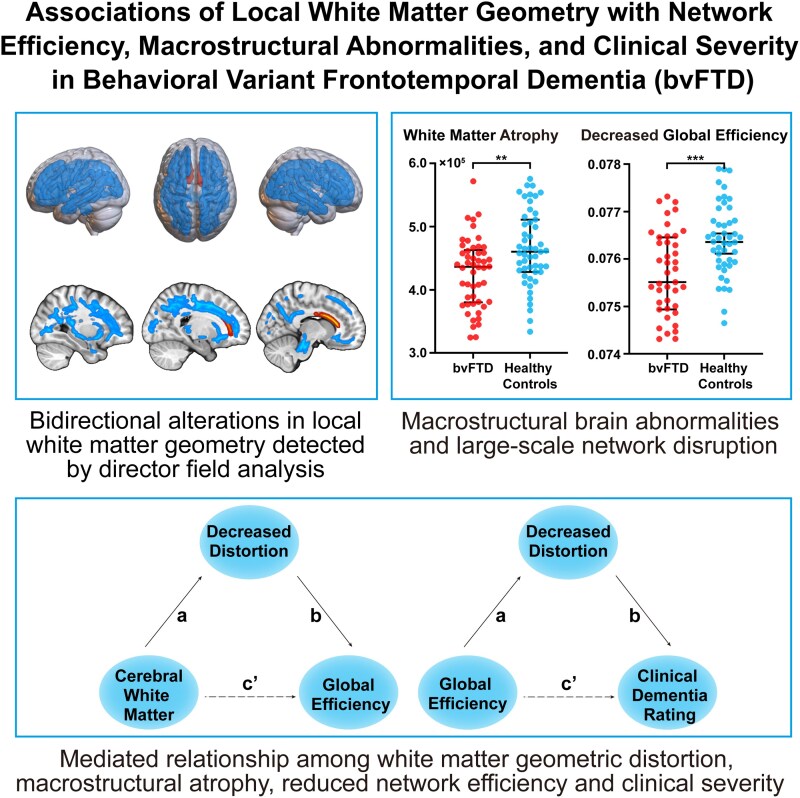

## Introduction

Frontotemporal dementia (FTD) is a common early-onset dementia, characterized by progressive atrophy of the frontal and/or temporal lobes of the brain.^1,2^ The behavioural variant of FTD (bvFTD), which presents with a spectrum of clinical manifestations such as personality changes, altered eating habits, deficits in empathy, and executive dysfunction, is the most common subtype of FTD.^1,3^ The precise neural mechanisms underlying bvFTD remain unknown but likely relate to the accumulation of misfolded proteins including tau, TAR-DNA-binding protein 43 (TDP-43), and fused in sarcoma (FUS).^4-6^ The accumulation and propagation of protein aggregates along axonal pathways may disrupt white matter (WM) integrity, which, along with other pathological factors, plays a crucial role in shaping the pattern of brain atrophy observed in bvFTD.^7-9^

Diffusion tensor imaging (DTI) is a non-invasive magnetic resonance imaging technique uniquely capable of quantifying the microstructural integrity and organization of WM pathways in vivo.^10,11^ In bvFTD, converging cross-sectional and longitudinal DTI studies have consistently demonstrated widespread reductions in fractional anisotropy (FA) and increases in mean diffusivity (MD), particularly within frontal and temporal association tracts^12-14^; these are thought to arise from axonal damage, demyelination, among others.^13,15-17^ While such changes in microstructure may be inherent to the neurodegenerative process, it is the local geometric orientation combined with global topological characteristics of WM fibres that may influence the propagation along axonal pathways, and thus the spatial trajectory of neurodegeneration. Using a novel director field analysis (DFA) method, a previous Alzheimer’s disease study revealed that WM geometric alterations exhibited neurodegeneration-related patterns not captured by conventional diffusion metrics such as FA and MD,^18^ highlighting the sensitivity of DFA-derived measures to fibre orientation and geometric organization beyond microstructural integrity alone.^19^ In addition to microstructural degeneration, macrostructural brain changes, most notably ventricular enlargement, are a prominent feature of bvFTD^20^ and may exert mechanical and physiological influences on the surrounding periventricular WM. Importantly, ventricular enlargement has also been suggested to reflect altered cerebrospinal fluid dynamics and impaired interstitial fluid clearance, processes closely linked to glymphatic system function.^21,22^ Such macrostructural and fluid-dynamic changes could contribute to alterations in WM fibre geometry, including orientation dispersion and spatial configuration. It is therefore likely that alterations in WM fibre geometry are closely coupled with macrostructural brain deformations and may, in turn, influence the efficiency of large-scale brain network communication, ultimately contributing to the functional deficits observed in bvFTD. However, to our knowledge, no studies have systematically characterized the local geometric properties of WM fibres in bvFTD. Elucidating these geometric alterations in bvFTD and their relationships with macrostructural brain deformation, network communication efficiency, and underlying biological processes may provide mechanistic insights into how multiscale structural reorganization contributes to large-scale network dysfunction and clinical manifestations of the disease.

The present study adopts an integrative, multi-level approach to investigate the role of WM geometric distortion in bvFTD. We examine changes in the local geometry of WM fibres and their associations with macrostructural brain morphological features, global network parameters, glymphatic function, and clinical data in patients with bvFTD (*n* = 5l) as compared to demographically matched healthy controls (*n* = 51, HCs). Specifically, we test a series of hypotheses that together form a coherent model: (1) Patients with bvFTD will exhibit regionally specific alterations in DFA metrics compared with HCs, reflecting prominent and widespread geometric changes of WM architecture. (2) Changes in DFA metrics will be associated with macrostructural morphological alterations (particularly lateral ventricular enlargement and cerebral grey matter atrophy) and with putative indicators of glymphatic dysfunction, as assessed non-invasively by the Diffusion Tensor Image Analysis along the Perivascular Space index. (3) Changes in DFA metrics will correlate with degradation of global network topology (e.g. reduced global efficiency), indicating that changes in the local geometry of WM fibres in bvFTD potentially influence the efficiency of information transfer within the large-scale brain networks. (4) Alterations in network efficiency will serve as a systems-level intermediate phenotype that not only reflects the impact of WM geometric changes on brain communication, but also will be linked to regional gene expression profiles via imaging-transcriptomic analyses, thereby helping to identify the biological substrates of geometry-related network dysfunction. Through this framework, we aim to clarify how the geometric properties of structural connections shape the progression of bvFTD, offering new perspectives on its pathophysiology.

## Materials and methods

### Ethics approval and consent to participate

Data used in the preparation of this article were obtained from the Frontotemporal Lobar Degeneration Neuroimaging Initiative (FTLDNI) database. The FTLDNI study was approved by the institutional review boards of all participating sites. All participants provided written informed consent prior to study participation according to the Declaration of Helsinki.

### Participants

Patients with bvFTD and HCs enrolled in the Frontotemporal Lobar Degeneration Neuroimaging Initiative (FTLDNI; http://memory.ucsf.edu/research/studies/nifd) were considered for inclusion in this study. The FTLDNI was funded by the National Institute on Aging (NIA) in 2010 to develop neuroimaging methods for tracking frontotemporal lobar degeneration (FTLD) and evaluate imaging biomarkers, which was led by Dr. Howard Rosen and involved three North American sites. The FTLDNI database is a multicentric database that collects positron emission tomography (PET), magnetic resonance imaging (MRI), and CSF biomarkers of patients with FTD and matched HCs. The FTLDNI study protocol involves a comprehensive multidimensional assessment of all participants, including clinical interviews, physical and neurological examinations, cognitive testing, biofluid sampling, and brain MRI scan. Specifically, patients with bvFTD were diagnosed using the Neary criteria,^23^ which integrate information from behavioural evaluation, cognitive profiles, and supportive features such as imaging. Their cognitive and clinical status was assessed using the Mini-Mental State Examination (MMSE) and the Clinical Dementia Rating (CDR) scales. Patients were excluded if they had major psychiatric or neurologic comorbidity, sustained hypertension, and vascular diseases. Healthy controls were required to have normal cognition (a CDR score of 0 and an MMSE score from 27 to 30), normal physical health and no prior history of mental illness. After excluding patients with poor image quality or incomplete image data, 51 patients with bvFTD and 51 HCs were included in this study.

### MRI data acquisition

The diffusion-weighted imaging (DWI) data were acquired on a 3.0T Siemens Tim Trio scanner equipped with a 12-channel head coil. The acquisition protocol employed a single-shot EPI sequence with the following parameters: anterior-posterior phase encoding, 44 diffusion directions, *b*-value = 0, 1000 s/mm^2^, repetition time (TR)/echo time (TE) = 9200/82 ms, slice thickness = 2.7 mm, and voxel size = 2.73 × 2.73 × 2.70 mm^3^. The T1-weighted MRI data were collected using a 3D MPRAGE sequence with the following parameters: TR/TE = 2300/2.98 ms, flip angle = 9°, slice thickness = 1 mm, and voxel size = 1 × 1 × 1 mm^3^.

### DFA analysis

Diffusion-weighted MRI data were preprocessed using the FMRIB Software Library (FSL, https://fsl.fmrib.ox.ac.uk/fsl), including corrections for eddy currents and head motion, and brain extraction. DMRITool (https://diffusionmritool.github.io) was then applied to compute diffusion tensor metrics (FA, MD) and four DFA metrics: bend, splay, twist, and total distortion.^19^ These DFA metrics capture three types of local orientational distortion geometry: splay (bending perpendicular to the fibre), bend (bending parallel to the fibre), and twist (rotational misalignment of neighbouring directions). The total distortion metric is a global measure of geometric distortion in WM tracts, which is defined as the square root of the sum of the squared bend, splay, and twist metrics. The detailed processing commands and critical parameters used for these computations are provided in the Supplementary Methods. Subsequently, WM tracts with significant alterations in these metrics were identified using the cross-species tractography (XTRACT) atlas^24-26^ in FSL and extracted using the tabulate function in MATLAB.

### Tract-based spatial statistics (TBSS) analysis

Voxel-wise analyses of FA, MD and four DFA metrics were conducted using the TBSS procedure in FSL.^27^ Specifically, all FA images were non-linearly registered to the Montreal Neurological Institute standard template space (MNI152).^28,29^ The normalized FA maps were averaged to create a group-wise mean FA map, which was then thresholded (FA > 0.2) to generate a mean FA skeleton. Subsequently, FA, MD, and four DFA metric values were projected onto the mean FA skeleton mask, generating skeletonized data for between-group comparisons. Between-group voxel-wise contrasts of these metrics were conducted with a non-parametric permutation test (5000 repetitions), adjusting for the confounding effects of age and gender. Threshold Free Cluster Enhancement (TFCE)^30^ was employed to correct for multiple comparisons and the level of significance was set at TFCE-corrected *P* < 0.05. Mean values of each of these metrics were then extracted from regions showing significant between-group differences.

### Morphological analysis

The T1-weighted images were analyzed using FreeSurfer (http://surfer.nmr.mgh.harvard.edu) with its standard preprocessing pipeline to derive the total cerebral white matter volume, total grey matter volume, total lateral ventricle volume, and total choroid plexus volume. Among them, the total lateral ventricle volume and total choroid plexus volume were determined by averaging the volumes of the corresponding structures across both hemispheres. Before conducting statistical analyses, the FreeSurfer outputs were visually checked to ensure the accuracy of relevant measurements, and participants with inadequate segmentation or surface reconstruction were excluded from the study. Differences in these morphological characteristics between patients with bvFTD and HCs were assessed using the Mann-Whitney Test.^31^

### Diffusion tensor image analysis along the perivascular space (DTI-ALPS)

The DTI-ALPS index was computed to evaluate the glymphatic function of each participant based on the DWI data.^32^ All DWI data were first preprocessed, including denoising, head motion and eddy-current correction. Subsequently, FA maps and direction-specific diffusivity maps (Dxx, Dyy, Dzz) were generated for each participant. These maps were then spatially normalized to the JHU-ICBM-FA template space^33,34^ using linear and non-linear registration algorithms in FSL. At the level of the lateral ventricular body, the anatomical locations corresponding to the projection fibres (superior corona radiata) and association fibres (superior longitudinal fasciculus) were defined using the JHU-ICBM-DTI-81-white-matter atlas. These predefined locations were then visually confirmed on the colour-coded FA maps, where these tracts consistently exhibit characteristic blue (superior-inferior) and green (anterior-posterior) colour coding, respectively. Four spherical regions of interest (ROIs) with 5-mm diameter were symmetrically drawn on these fibres to create masks, which were then applied to all participants’ diffusivity maps. Finally, the DTI-ALPS index was computed as a ratio of (Dxx*proj* + Dxx*assoc*)/(Dyy*proj* + Dzz*assoc*),^32^ where Dxx*proj* and Dyy*proj* represent the mean diffusivities along the *x*- and *y*-axes in projection fibres, while Dxx*assoc* and Dzz*assoc* denote the mean diffusivities along the *x*- and *z*-axes in association fibres, respectively. All intermediate processing outputs were visually checked to ensure data integrity and proper spatial alignment. Any images with poor quality or evidence of processing errors were identified and excluded.

### Large-scale WM network analysis

The DWI data were preprocessed using FSL for denoising, eddy current and head motion correction. WM fibre tractography and structural network construction were performed in MRtrix3 (v3.0.3; https://www.mrtrix.org/). Single-shell multi-tissue constrained spherical deconvolution (CSD) was applied to obtain fibre orientation distributions (FODs), followed by whole-brain tractography using the iFOD2 algorithm with dynamic seeding (FOD cutoff = 0.06, 10 million streamlines, backtracking enabled). The network nodes were defined using the Desikan-Killiany (DK) atlas^35,36^ from FreeSurfer. The tck2connectome tool was used to calculate interregional streamline counts for constructing an 84 × 84 symmetric connectivity matrix. The matrix was normalized to edge density with diagonal elements excluded to avoid self-connections. To ensure data quality, probabilistic streamline filtering was applied to retain over 90% of high-confidence fibres, followed by visual inspection of major white matter tracts to verify anatomical plausibility.

In this study, four global network parameters, including global efficiency (Eg), local efficiency (Eloc), shortest path length (Lp), and clustering coefficient (Cp), were calculated using the GRETNA Toolbox in MATLAB. These parameters reflect key characteristics of brain network architecture, such as integration, segregation, information transfer speed, and modularity.^37,38^ Specifically, Eg quantifies the efficiency of parallel information transfer across the entire network and reflects the level of global integration.^39,40^ Eloc measures the efficiency of information exchange within local neighbourhoods, representing functional segregation and fault tolerance.^39,40^ Lp reflects the global efficiency (or the typical minimum number of steps) of information transfer across the network, while Cp captures the tendency of nodes to form locally interconnected clusters, indicating the degree of network segregation and modular organization.^39,41^ In addition to global metrics, nodal efficiency, which reflects how efficiently a given node communicates with all other nodes in the network, was calculated to assess the contribution of individual brain regions to global information integration.^39,40^ Detailed mathematical definitions and computational formulas of these network parameters can be found in the Supplementary Methods. Subsequently, a sparsity-based thresholding strategy to minimize selection bias was adopted. For each participant, the adjacency matrix was binarized by preserving the strongest S% of edges, thereby maintaining consistent edge counts for group comparisons. In this study, network parameters were computed over a sparsity range of 0.10 to 0.25 (step size = 0.01) for all participants. Finally, the Mann-Whitney tests were employed to compare the network parameters between the two groups using the area under the curve (AUC) values across the sparsity range. For the nodal parameter, the results were further adjusted for multiple comparisons using the false discovery rate (FDR) method.

### Neuroimaging-transcriptional association analysis

A neuroimaging-transcriptional association analysis was performed to explore the potential biological processes accounting for the between-group nodal efficiency deviances. This analysis aimed to identify gene expression patterns co-varying with regional brain network alterations in bvFTD. First, the average between-group difference in nodal efficiency was computed for each of the 84 regions defined by the DK Atlas, resulting in an 84 × 1 vector representing the nodal efficiency deviations. Next, gene expression data were obtained from the Allen Human Brain Atlas (AHBA) transcriptomic dataset and mapped onto the 84 regions of the DK Atlas using the abagen toolbox (https://github.com/rmarkello/abagen).^42,43^ This process created an 84 × 16 533 regional transcription matrix. Then, a partial least squares (PLS) regression was performed to explore the relationship between regional transcription data and the nodal efficiency deviance vector, with the regional transcription data as the predictor variables and the nodal efficiency deviance vector as the response variable. The PLS1 represents the linear combination weights of gene expression that best predict the neuroimaging feature across regions. A permutation test with 1000 replications was conducted to assess the statistical significance of PLS1. The error related to each gene’s PLS1 weight was estimated via bootstrapping, resampling the regions the 84 regions 1000 times with replacement. Subsequently, a Z-score was calculated for each gene as the ratio of the weight to its bootstrap standard deviation. Genes with a |Z-score| > 3, indicative of significant nodal efficiency deviances between patients with bvFTD and HCs, were selected for downstream analysis. Finally, the list of significant genes was used for enrichment analysis using Metascape (https://metascape.org/) to identify enrichments in Gene Ontology (GO) biological processes and Kyoto Encyclopedia of Genes and Genomes (KEGG) pathways. Terms with a corrected *P*-value < 0.05 were considered statistically significant.

### Correlation and mediation analyses among imaging and clinical data

Both Pearson’s and Spearman’s correlation coefficients were assessed to examine the associations among the DFA metrics, clinical data, morphological metrices, DTI-ALPS index and global network parameters. The level of significance was determined using two-tailed tests with a threshold of *P* < 0.05. Here, Pearson’s correlation coefficient was primarily used for normally distributed data, while Spearman’s correlation coefficient was employed when the normality assumption was violated (assessed via Shapiro-Wilk tests).

Mediated relationships between imaging and clinical measures in bvFTD were examined using the ‘mediation toolbox’ in MATLAB,^44^ with age and gender included as covariates to control for potential confounding effects. Prior to analysis, all variables were standardized to z-scores to ensure scale-invariant interpretation of coefficients. Bootstrap mediation analysis with 10 000 iterations was conducted to estimate the potential mediation effects.

## Results

### Demographic and clinical characteristics

A total of 51 patients with bvFTD and 51 HCs were included in this study (Table 1). There was no significant difference in age (*P* = 0.5666) and sex composition (*P* = 1) between the two groups. Patients with bvFTD had higher CDR scores (*P* < 0.0001) and lower MMSE scores (*P* < 0.0001) than HCs.

**Table 1:** Demographic and clinical data

Variables	bvFTD	HCs	*P*-value
Number of subjects	51	51	
Gender (male/female)	35/16	35/16	1.0000
Age (years)	60.78 ± 6.35	60.98 ± 6.48	0.5666
CDR scores	5.85 ± 3.28	0.09 ± 0.23	<0.0001
MMSE scores	24.29 ± 4.64	29.33 ± 0.77	<0.0001

**Note:** All data are represented as mean ± standard deviation. Between-group differences were examined using two-sample *t*-test and χ^2^ test.

**Abbreviations:** bvFTD, behavioural variant frontotemporal dementia; CDR, Clinical Dementia Rating; HCs, healthy controls; MMSE, Mini-Mental State Examination.

### WM microstructural changes in bvFTD

**Figure 1:**
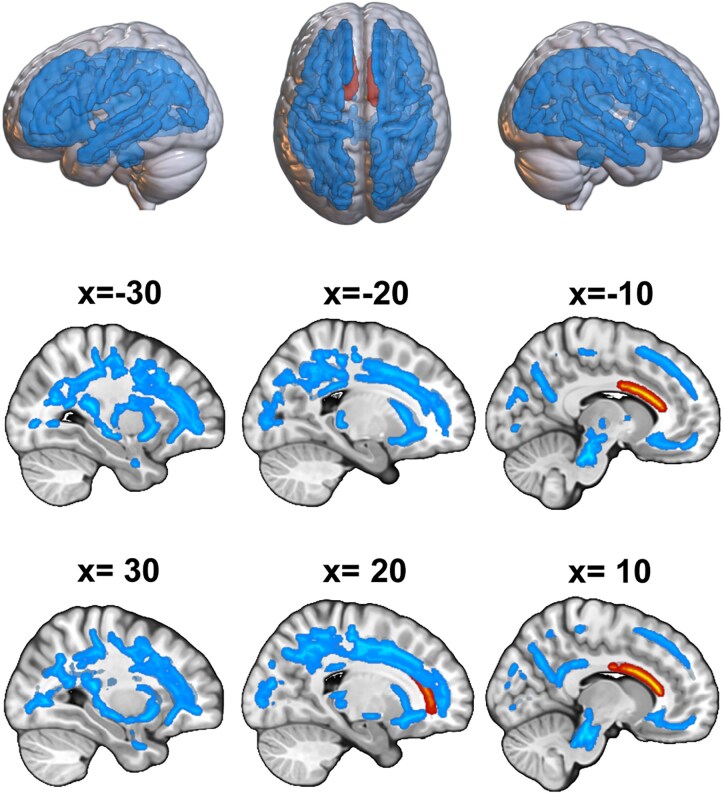
**Brain regions showing significant differences in total distortion between patients with bvFTD (*N* = 51) and HCs (*N* = 51).** Between-group comparisons were performed using a non-parametric permutation test (5000 repetitions), adjusting for age and gender. Cool colours indicate regions where patients with bvFTD had significantly lower distortion compared with HCs, while warm colours indicate regions with significantly higher distortion in patients with bvFTD than in HCs. The result was obtained with a threshold of TFCE-corrected *P* < 0.05.

Patients with bvFTD exhibited significant bidirectional alterations in the local geometric characteristics of WM fibres. Compared with HCs, patients with bvFTD showed increased geometric distortion in the forceps minor (FMI) and dorsal cingulum, alongside decreased geometric distortion in the inferior fronto-occipital fasciculus (IFOF), superior longitudinal fasciculus (SLF), anterior thalamic radiation (ATR), uncinate fasciculus (UF), frontal aslant tract (FAT), and arcuate fasciculus (AF) (Fig. 1). Further analysis on three specific geometric distortion measures showed increased splay, bend, and twist in the FMI and dorsal cingulum, along with a differential pattern of decreases in widespread WM regions (Supplementary Fig. 1). Specifically, splay reductions were observed in the FAT, ATR, SLF, UF, IFOF and superior thalamic radiation (STR) (Supplementary Fig. 1A). Bend reductions were found in the ATR, FMI, SLF, UF, IFOF and FAT (Supplementary Fig. 1B). Twist reductions were located in the IFOF, SLF, ATR, middle longitudinal fasciculus (MLF), FAT and UF (Supplementary Fig. 1C). These DFA findings contrasted with the unidirectional decrease in FA and increase in MD in these patients (Supplementary Fig. 2), suggesting that the DFA metrics carry unique information, and their changes are not just a neurodegenerative sequela affecting WM microstructure.

### Morphological and glymphatic changes in bvFTD

**Figure 2:**
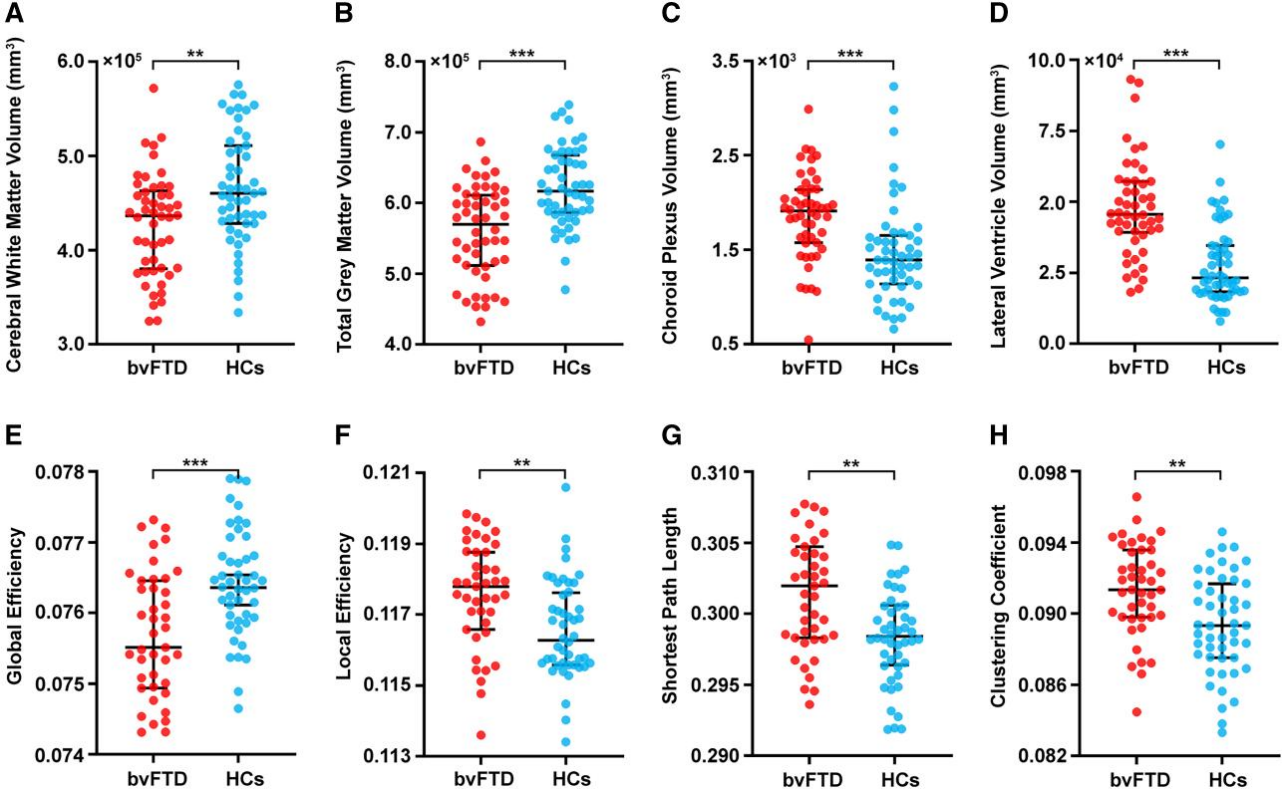
**Differences in macrostructural brain morphological features and global network parameters between patients with bvFTD (*N* = 51) and HCs (*N* = 51). (A)** cerebral white matter, **(B)** total grey matter, **(C)** choroid plexus, **(D)** lateral ventricle, **(E)** global efficiency, **(F)** local efficiency, **(G)** shortest path length and **(H)** clustering coefficient in patients with bvFTD were compared with those in HCs using a Mann-Whitney Test. Individual data points shown on the plots represent a single participant. All data are presented as median (25% fraction, 75% fraction); * denotes *P* < 0.05, ** denotes *P* < 0.01, *** denotes *P* < 0.001.

Compared with HCs, patients with bvFTD showed significantly decreased volumes of cerebral white matter (*P* = 0.0026, Fig. 2A) and total grey matter (*P* < 0.0001, Fig. 2B). In contrast, they exhibited significantly enlarged choroid plexus (*P* < 0.0001, Fig. 2C) and lateral ventricles (*P* < 0.0001, Fig. 2D). Moreover, patients with bvFTD showed decreased DTI-ALPS values (*P* < 0.0001), indicating putative glymphatic dysfunction (Supplementary Table 1).

### WM network topological changes in bvFTD

Compared with HCs, patients with bvFTD showed significantly decreased global efficiency (Eg) and increased local efficiency (Eloc), along with longer shortest path length (Lp) and higher clustering coefficient (Cp) across several network sparsity levels (0.1–0.25, Fig. 2E–H, Supplementary Fig. 3 and Supplementary Table 2). Subsequent area under the curve (AUC) analyses, which integrate effects across multiple sparsity levels, further confirmed these findings that patients with bvFTD demonstrated significantly lower Eg (*P* = 0.0010, Fig. 2E) and higher Eloc (*P* = 0.0014, Fig. 2F), alongside higher Lp (*P* = 0.0015, Fig. 2G) and Cp (*P* = 0.0011, Fig. 2H) (Supplementary Table 1), reflecting an overall reduction in global integration but higher local segregation in the network.

**Figure 3:**
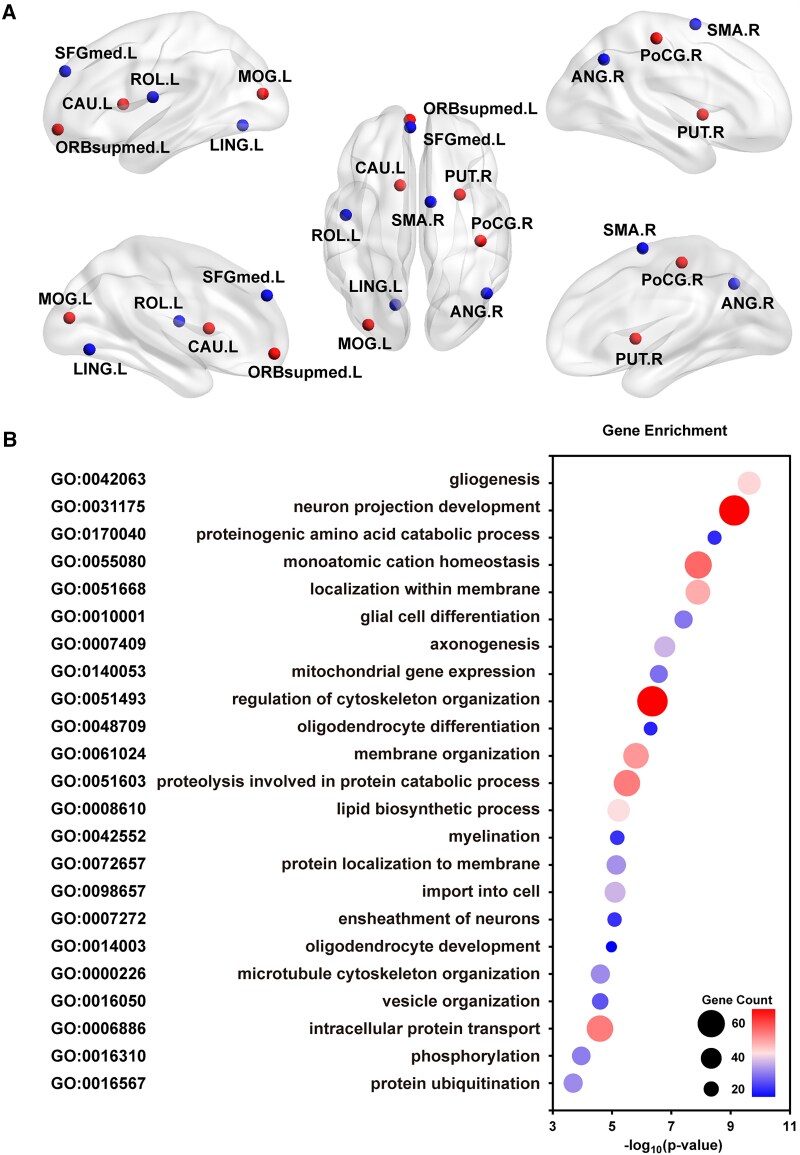
**Between-group differences in nodal efficiency and their transcriptomic bases. (A)** Brain regions with significant differences in nodal efficiency between patients with bvFTD (*N* = 51) and HCs (*N* = 51). Between-group comparisons were performed using non-parametric permutation testing. Red nodes represent brain regions where patients with bvFTD showed significantly higher nodal efficiency compared with HCs, while blue nodes indicate regions with significantly lower nodal efficiency in patients with bvFTD relative to HCs. The results were obtained with a threshold of FDR-corrected *P* < 0.05. **(B)** Functional enrichment analysis of the pivotal genes associated with the nodal efficiency deviances between patients with bvFTD and HCs. Terms were retained with a threshold of FDR-corrected *P* < 0.05. ORBsupmed.L, left medial orbital superior frontal gyrus; CAU.L, left caudate nucleus; MOG.L, left middle occipital gyrus; PoCG.R, right postcentral gyrus; PUT.R, right putamen; SFGmed.L, left medial superior frontal gyrus; ROL.L, left Rolandic operculum; LING.L, left lingual gyrus; SMA.R, right supplementary motor area; ANG.R, right angular gyrus.

Nodal efficiency analysis revealed significant bidirectional alterations across several brain regions in bvFTD. Specifically, patients with bvFTD showed decreased nodal efficiency in the left medial superior frontal gyrus, left lingual gyrus, left Rolandic operculum, right angular gyrus and right supplementary motor area, while showing increased nodal efficiency in the left middle occipital gyrus, left medial orbital superior frontal gyrus, left caudate nucleus, right putamen and right postcentral gyrus (Fig. 3A).

### Neuroimaging-transcriptional association analysis

To investigate the biological processes/pathways underlying the nodal efficiency differences between patients with bvFTD and HCs, we conducted a neuroimaging-transcriptional association analysis using the Allen Human Brain Atlas (AHBA). Through partial least squares regression (PLS), we identified a significant association between gene expression patterns and nodal efficiency alterations. Specifically, the first PLS component (PLS1), calculated as the weighted sum of the 1895 gene expression profiles (|Z-score| > 3), showed a significant association with the nodal efficiency differences, accounting for 28.24% of the variances (permuted *P* = 0.003). Functional enrichment analysis of the identified gene list revealed a number of biological processes/pathways highly relevant to the formation and transport of TDP-43 and/or tau aggregates along axonal pathways (such as ‘intracellular protein transport’, ‘protein localization to membrane’, ‘phosphorylation’, and ‘microtubule cytoskeleton organization’, among others) and those tightly linked to disturbances in cellular homeostasis that may exacerbate the pathology of TDP-43 and/or tau aggregates (such as ‘monoatomic cation homeostasis’, ‘lipid biosynthetic process’, and ‘proteolysis involved in protein catabolic process’) (Fig. 3B). These findings suggest that nodal efficiency alterations in bvFTD are mechanistically tied to (1) impaired axonal transport of pathological proteins and (2) systemic breakdowns in cellular homeostasis, which collectively accelerate neurodegeneration.

### Relationships among imaging and clinical data

**Figure 4:**
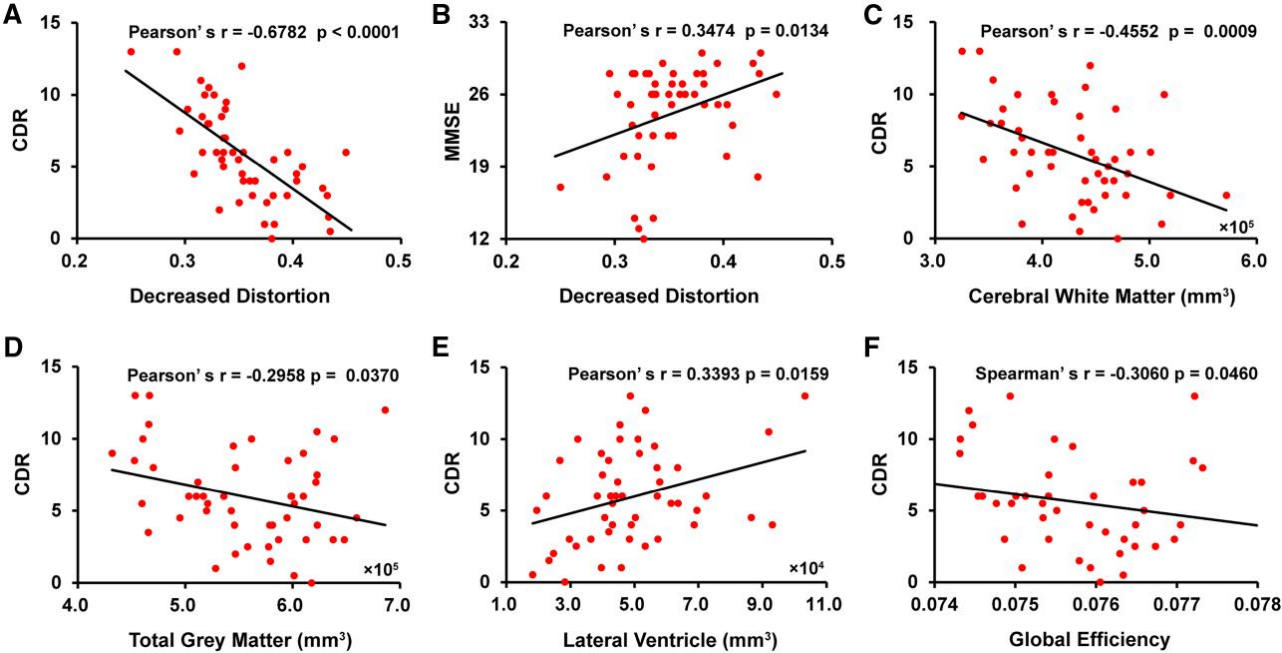
**Associations between imaging and clinical data in patients with bvFTD (*N* = 51).** Correlations between distortion decrease and CDR score **(A)**, distortion decrease and MMSE score **(B)**, cerebral white matter volume and CDR score **(C)**, total grey matter volume and CDR score **(D)**, lateral ventricle volume and CDR score **(E)**, and global efficiency and CDR score **(F)**. Correlation analyses were performed using Pearson’s or Spearman’s correlation, as described in the Methods section. Each data point represents a single patient.

In patients with bvFTD, the decrease in geometric distortion was negatively correlated with CDR scores (*r* = −0.6782, *P* < 0.0001, Fig. 4A) and positively correlated with the MMSE scores (*r* = 0.3474, *P* = 0.0134, Fig. 4B). Additionally, decreased volumes of cerebral white matter (*r* = −0.4552, *P* = 0.0009, Fig. 4C) and total grey matter (*r* = −0.2958, *P* = 0.0370, Fig. 4D) were negatively correlated with CDR scores, whereas enlarged lateral ventricles positively correlated with CDR scores (*r* = 0.3393, *P* = 0.0159, Fig. 4E). Meanwhile, ventricular enlargement (*r* = −0.3653, *P* = 0.0091) and choroid plexus expansion (*r* = −0.3394, *P* = 0.0159) were associated with poorer MMSE performance (Supplementary Table 3). Reduced global efficiency was negatively correlated with CDR scores (*r* = −0.3060, *P* = 0.0460, Fig. 4F).

**Figure 5:**
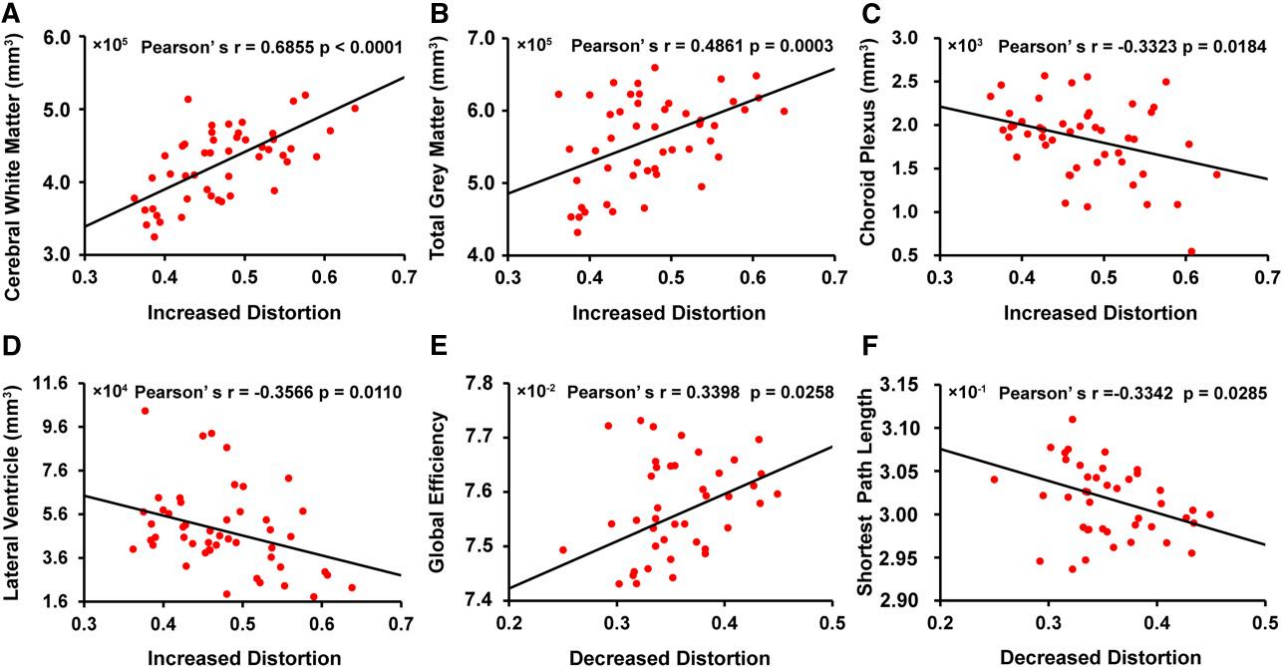
**Associations of geometric distortion alterations with morphological and network parameters in patients with bvFTD (*N* = 51).** Correlations between distortion increase and cerebral white matter volume **(A)**, distortion increase and total grey matter volume **(B)**, distortion increase and choroid plexus volume **(C)**, distortion increase and lateral ventricle volume **(D)**, distortion decrease and global efficiency **(E)**, distortion decrease and shortest path length **(F)**. Correlation analyses were performed using Pearson’s or Spearman’s correlation, as described in the Methods section. Each data point represents a single patient.

For the intermodal correlation analysis, reductions in cerebral white matter volume (*r* = 0.6855, *P* < 0.0001, Fig. 5A) and total grey matter volume (*r* = 0.4861, *P* = 0.0003, Fig. 5B) were positively correlated with the increase in distortion. Increases in the choroid plexus (*r* = −0.3323, *P* = 0.0184, Fig. 5C) and lateral ventricle volumes (*r* = −0.3566, *P* = 0.011, Fig. 5D) were negatively correlated with the increase in distortion. Enlargement of the lateral ventricles was associated with diminished glymphatic activity as measured by DTI-ALPS (*r* = −0.6020, *P* < 0.0001). Meanwhile, the decrease in distortion was positively correlated with a reduction in global efficiency (*r* = 0.3398, *P* = 0.0258, Fig. 5E) and negatively correlated with an increase in path length (*r* = −0.3342, *P* = 0.0285, Fig. 5F), suggesting a critical role of WM geometry in maintaining global network integration. For more detailed associations between DFA metrics and other imaging/clinical data, please refer to Supplementary Table 4.

**Figure 6:**
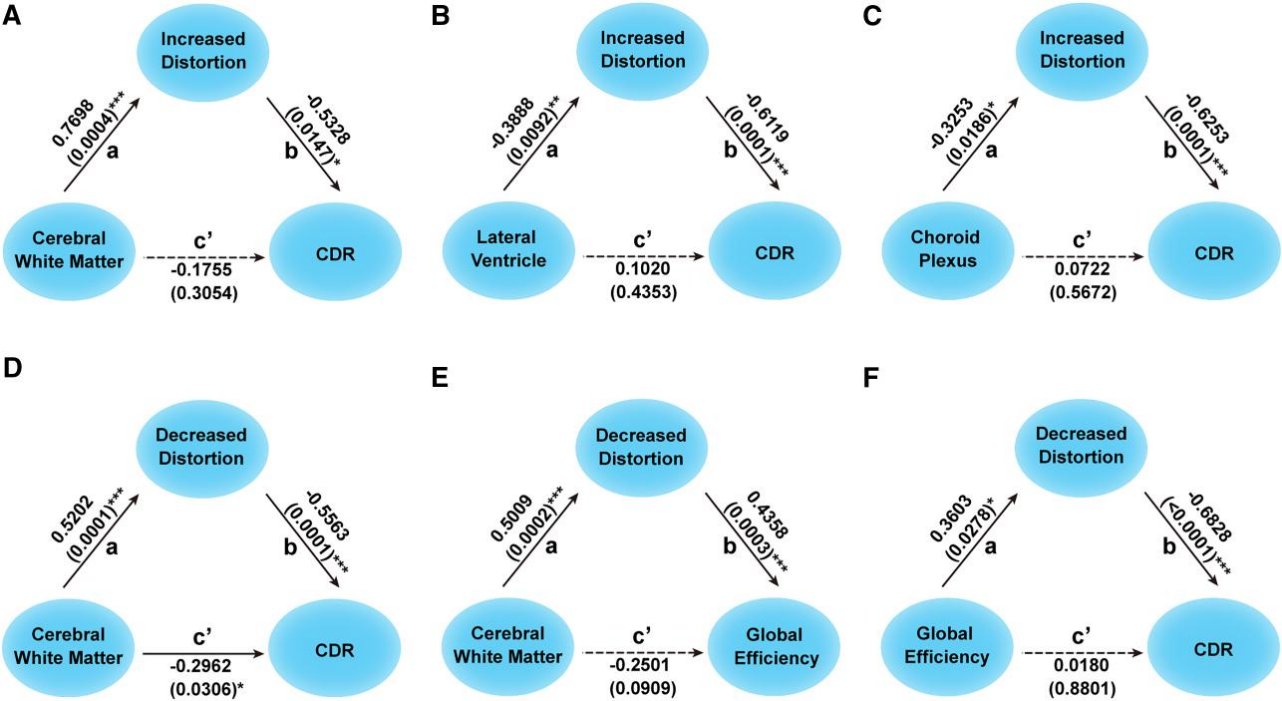
**Mediation analyses among imaging and clinical data in patients with bvFTD.** Bootstrap mediation analysis with 10 000 iterations was conducted to estimate the potential mediation effects, with age and gender included as covariates. All variables were standardized to *z*-scores to ensure scale-invariant interpretation of coefficients. In each model diagram (**A–F**), path coefficients are labelled a (X→M), b (M→Y), and c′ (X→Y). For each path, the unstandardized coefficient is displayed, followed by the *P*-value in parentheses; solid and dashed arrows denote significant and non-significant paths, respectively. * denotes *P* < 0.05, ** denotes *P* < 0.01, *** denotes *P* < 0.001.

To investigate the mediating role of geometric distortion in linking macrostructure, network efficiency, and functional impairments, we conducted a series of mediation analyses in patients with bvFTD. We found that geometric distortion alterations significantly mediated the effects of macrostructural abnormalities on disease severity. Specifically, increased distortion mediated the associations of reduced cerebral white matter volume, enlarged lateral ventricles, and choroid plexus expansion with higher CDR scores (Fig. 6A–C), while decreased distortion mediated the associations of reduced cerebral white matter volume with both reduced global efficiency and higher CDR scores (Fig. 6D, E). Besides, reduced distortion also mediated the relationship between global efficiency decline and higher CDR scores (Fig. 6F).

## Discussion

Utilizing a novel DFA method, this study revealed bidirectional alterations in local geometric distortions, including abnormal bending, splaying, and twisting of WM fibres, in patients with bvFTD. These microstructural geometric changes were associated with macrostructural abnormalities (e.g. grey and white matter atrophy, as well as ventricular and choroid plexus enlargement), marked glymphatic dysfunction and reduced global network efficiency in bvFTD. More intriguingly, they significantly mediated the relationship between macrostructural abnormalities, network inefficiency, and clinical severity. Additionally, the nodal efficiency changes were associated with TDP-43/tau pathologies along axonal pathways, and cellular homeostasis disturbances, as revealed by neuroimaging-transcriptomic association analysis. Together, these findings suggest that the local geometric alterations of WM fibres may be an endophenotypic manifestation of both proteinopathy-driven neurodegeneration and adaptive processes in response to worsening proteinopathy.

The bidirectional nature of WM local geometric distortions, marked by increased distortion in the FMI and dorsal cingulum, alongside decreased distortion in IFOF, SLF, ATR, UF, FAT, and AF, highlights a spatially stratified pattern of axonal disruption in bvFTD. These WM fibres are pivotal to clinical deficits in bvFTD. Hyperdistortion in the FMI, a key component of the frontoparietal control network, is associated with disinhibition and executive dysfunction.^16,45^ In contrast, hypodistortion in the IFOF and UF, which interconnect orbitofrontal and anterior temporal regions, potentially leads to deficits in emotional regulation and social cognition (e.g. apathy and disinhibition).^16,46,47^ Additionally, abnormalities in the SLF may contribute to working memory dysfunction,^48,49^ while disruptions in the FAT and ATR are more likely associated with executive function deficits.^49,50^ Similarly, damage to the AF could negatively impact speech and language processing.^51^ The negative correlation between CDR scores and reduced geometric distortion in these tracts further underscores their clinical relevance. Notably, these bidirectional alterations in WM local geometry contrasted with unidirectional microstructural changes captured by traditional DTI metrics observed in the same cohort (e.g. decreased FA and increased MD). This divergence likely arises because DTI metrics reflect broad microstructural degradation (e.g. axonal loss, demyelination) through water diffusion patterns, whereas DFA directly quantifies localized fibre architecture disruptions, such as axonal compression (hyperdistortion) or Wallerian degeneration (hypodistortion). By mapping these WM geometric abnormalities to specific clinical phenotypes, this study establishes the first attempt to characterize the local geometry of WM fibre distortion as a novel biomarker capturing both degenerative and adaptive processes in bvFTD. Our findings challenge the traditional view of WM pathology as a uniform disconnection syndrome, instead revealing a dynamic interplay of localized architectural collapse and maladaptive plasticity in WM local geometry that drives functional decline in bvFTD.

Our analyses revealed critical links between macrostructural morphological alterations and bidirectional changes of geometric distortion in WM fibres in bvFTD. Patients exhibited reduced total grey and white matter volumes alongside enlarged choroid plexus and lateral ventricles, which all correlated strongly with increased geometric distortion. These findings align with prior reports of choroid plexus hypertrophy and ventricular expansion as markers of progressive WM atrophy and Cerebrospinal Fluid (CSF) dysregulation in bvFTD.^21,52^ Mechanistically, choroid plexus enlargement may drive excessive CSF production, exacerbating ventricular dilation.^53^ This expansion could exert mechanical stress on periventricular WM tracts (e.g. corpus callosum, fornix), compressing or displacing fibres—a hypothesis supported by the proximity of hyperdistorted WM regions to the ventricles. Notably, increased geometric distortion mediated the relationship between WM volume loss and functional decline (measured by CDR scores), positioning WM geometric disruption as a conduit through which structural atrophy translates into clinical disability. This mediation implies that WM degeneration in bvFTD is not merely a passive loss but an architecturally disruptive process, where axonal disorganization impairs network efficiency.

Further analyses revealed that bidirectional changes in local geometric distortions of WM fibres are linked to global network disintegration and compensatory local reorganization in bvFTD. Patients exhibited decreased global efficiency and increased local efficiency, accompanied by longer characteristic path lengths and higher clustering coefficients, reflecting a shift toward fragmented, locally segregated networks at the expense of global integration. Specifically, reduced geometric distortion correlated strongly with global efficiency declines, suggesting that the loss of long-range fibre integrity (e.g. straightened SLF, IFOF and UF) undermines information communication across distributed regions. Conversely, increased geometric distortion (e.g. splayed FMI and dorsal cingulum) showed no significant association with global efficiency. This dissociation highlights that global network collapse in bvFTD is driven predominantly by widespread WM hypodistortion (reduced fibre complexity), which diminishes the brain’s capacity to integrate information across distributed hubs. More interestingly, mediation analyses revealed that decreased geometric distortion regulate the association between global efficiency loss and functional decline, suggesting WM degeneration may amplify network disintegration into clinical disability, as atrophic tracts (e.g. FAT, AF) impair information transmission through critical hubs like the medial superior frontal gyrus and angular gyrus.^54,55^ Moreover, increased nodal efficiency in non-hub regions (e.g. caudate, postcentral gyrus) may reflect maladaptive plasticity, where preserved circuits hyperactivate to compensate for hub disconnection. This aligns with the hub-priority damage model,^56^ where targeted hub degradation destabilizes global efficiency, while non-hub compensatory changes have minimal restorative impact. These findings collectively characterize bvFTD as involving a dual mechanism of structural network collapse and ineffective local compensation, wherein bidirectional geometric distortions in white matter fibres serve as a structural substrate driving and mirroring both processes.

Neuroimaging-transcriptional association analysis revealed that nodal efficiency alterations in bvFTD were linked to the formation and spread of TDP-43 and/or tau aggregates. Specifically, dysregulations in protein transport, such as ‘intracellular protein transport’, ‘protein localization’ and ‘localization within membrane’, are key drivers of the mislocalization and accumulation of both the predominantly nuclear RNA-binding protein TDP-43 and the microtubule-associated protein tau.^57-59^ The ‘phosphorylation’ and ‘ubiquitination’ processes, as the most common post-translational modifications, could further impact their activity, stability, localization, and aggregation propensity.^60,61^ Indeed, studies have revealed that TDP-43 and tau deposit and aggregate along axons, spreading to neighbouring cells and triggering axonal degeneration and neuronal apoptosis.^7,62^ Meanwhile, the identified set of genes was also enriched in ‘lipid biosynthetic process’, ‘proteolysis involved in the protein catabolic process’ and ‘monoatomic cation homeostasis’, whose dysregulation may trigger oxidative stress and induce neuroinflammation.^63,64^ For example, abnormalities in the ‘monoatomic cation homeostasis’ process may disrupt cellular osmotic balance and ion gradients, potentially impairing AQP4-mediated water transport.^65,66^ This impairment could affect CSF flow and substance exchange, leading to glymphatic system dysfunction and the accumulation of metabolic waste in brain tissue.^67,68^ These accumulated waste products are likely to activate microglia and astrocytes, prompting them to release pro-inflammatory factors, thereby triggering a neuroinflammatory response.^69,70^ The inflammatory state, in turn, can further exacerbate the overactivation of glial cells and the dysfunction of the glymphatic system, compromising their ability to clear pathogenic aggregates.^57,71,72^ Consequently, this disruption might create a vicious cycle, worsening the inefficiency in clearing pathogenic proteins and fuelling further neuroinflammation. Furthermore, abnormalities in ‘glial cell differentiation’, particularly in ‘oligodendrocyte differentiation’, may have negative effects on remyelination, while the neuroinflammatory environment mentioned above might further damage the development and function of oligodendrocytes, potentially resulting in demyelination and alterations in WM microstructure.^73,74^ Taken together, these findings indicate that the observed efficiency decline in bvFTD might be a macroscopic manifestation of WM microstructural abnormalities resulting from disrupted biological processes or pathways crucial for the development and propagation of TDP-43 and/or tau pathologies.

### Limitations

The present study has several limitations that should be acknowledged. First, the relatively modest sample size may limit the generalizability of our findings regarding the local geometry of WM fibres in bvFTD. Replication in larger, independent cohorts is warranted. Second, since mediation analysis can only establish associations between variables and cannot directly infer causal relationships, the interpretation of the relevant results in this study remains somewhat speculative and therefore should be approached with caution. Third, the DFA analysis was restricted to the TBSS skeleton, which, while standard for whole-brain comparison, may fail to fully capture microstructural alterations in deeper portions of white matter tracts. Future studies employing tract-specific analyses along tractography-derived pathways could complement these findings by providing more anatomically precise profiles. Fourth, the biological processes associated with WM geometric alterations in bvFTD were indirectly inferred through their relationship with network efficiency, rather than representing mechanisms specifically tied to WM geometry itself. Future studies are warranted to further clarify this issue.

## Conclusion

The present study revealed significant bidirectional changes in the local geometry of WM tracts in patients with bvFTD. The WM microstructural alterations not only mediated the relationship between macrostructural brain morphological changes and cognitive impairments but also demonstrated a strong association with a reduction in global network efficiency. These findings may contribute to the understanding of the neural bases underlying the functional impairments in bvFTD and suggest potential mechanistic pathways for developing innovative therapeutic strategies for this disorder.

## Supplementary Material

fcag226_Supplementary_Data

## Data Availability

Data used in the preparation of this article were obtained from the Frontotemporal Lobar Degeneration Neuroimaging Initiative database (https://ida.loni.usc.edu/collaboration/access/appLicense.jsp). The preprocessing of diffusion-weighted MRI images and TBSS analysis were performed using FSL (https://fsl.fmrib.ox.ac.uk/fsl). FA, MD, and four DFA metrics were computed using DMRITool (https://diffusionmritool.github.io). T1-weighted images were processed with FreeSurfer (http://surfer.nmr.mgh.harvard.edu). The DTI-ALPS index was calculated using the tools provided in the open-source repository (https://github.com/gbarisano/alps). Large-scale WM network analysis was conducted using FSL, MRtrix3 (https://www.mrtrix.org/), FreeSurfer, and the GRETNA Toolbox in MATLAB (https://www.nitrc.org/projects/gretna/). Enrichment analysis was conducted via Metascape (https://metascape.org). The statistical analyses including correlation analysis, two-sample *t*-test, χ^2^ test and Mann-Whitney U tests were computed using GraphPad Prism 9 (https://www.graphpad.com). Mediation analyses were conducted using the Mediation Toolbox in MATLAB (https://github.com/canlab/MediationToolbox).

## Appendix

FTLDNI investigators: Howard Rosen, Bradford C. Dickerson, Kimoko Domoto-Reilly, David Knopman, Bradley F. Boeve, Adam L. Boxer, John Kornak, Bruce L. Miller, William W. Seeley, Maria-Luisa Gorno-Tempini, Scott McGinnis, Maria Luisa Mandelli.
