# Supplementary material for: Associations of local white matter geometry with network efficiency, macrostructural abnormalities, and clinical severity in behavioural variant frontotemporal dementia
Source: Brain Commun. 2026 Jun 16;8(3):fcag226. doi: 10.1093/braincomms/fcag226 (PMC13297003; doi:10.1093/braincomms/fcag226)
Supplement: fcag226_Supplementary_Data [file fcag226_supplementary_data.docx]

**Associations of Local White Matter Geometry with Network Efficiency, Macrostructural Abnormalities, and Clinical Severity in Behavioral Variant Frontotemporal Dementia**

**Short title**: Local WM Geometry in bvFTD

Qinyao Sun, MSc^1, 2,^ †, Yu Zhang, PhD^3,^ †, Xin Jin, MSc^1, 2,^ †, Jian Cheng, PhD^4,^ †, Jianyu Li, MSc^1, 2^, Ting Qiu, PhD^5^, Zhanbing Ren, PhD^6^, Ke Li, PhD^1, 2^, Huixiong Zhang, PhD^1, 2^, For the Frontotemporal Lobar Degeneration Neuroimaging Initiative, Kewei Chen, PhD^7^, Lena Palaniyappan, PhD^5^, Yifan Chen, PhD^1, 2,^ *, B. Blair Braden, PhD^7,^ *, Yuanchao Zhang, PhD^1, 2, 7,^ *

^1^The Clinical Hospital of Chengdu Brain Science Institute, MOE Key Lab for Neuroinformation, University of Electronic Science and Technology of China, Chengdu, 611731, P. R. China

^2^School of Life Science and Technology, University of Electronic Science and Technology of China, Chengdu, 610054, Sichuan, P. R. China

^3^School of Psychology, Shanghai Jiao Tong University, Shanghai, 200030, China

^4^School of Computer Science and Engineering, Beihang University, Beijing, China

^5^Douglas Mental Health University Institute, McGill University, Montreal, Canada

^6^College of Physical Education, Shenzhen University, Shenzhen, P. R. China

^7^College of Health Solutions, Arizona State University, Phoenix, AZ, USA

†Qinyao Sun, Yu Zhang, Xin Jin and Jian Cheng contributed equally to this work.

Correspondence to:

**Yuanchao Zhang**

College of Health Solutions,

Arizona State University, Lattie F. Coor Hall, 976 S Forest Mall, Tempe, AZ 85281, USA

E-mail: yuanchao.zhang@asu.edu or [yuanchao.zhang8@gmail.com](mailto:yuanchao.zhang8@gmail.com)

Correspondence may also be sent to:

**Yifan Chen**

School of Life Science and Technology,

University of Electronic Science and Technology of China, Chengdu, 610054, Sichuan, P. R. China

E-mail: [yifan.chen@uestc.edu.cn](mailto:yifan.chen@uestc.edu.cn)

**B. Blair Braden**

College of Health Solutions,

Arizona State University, Lattie F. Coor Hall, 976 S Forest Mall, Tempe, AZ 85281, USA

E-mail: [bbbraden@asu.edu](mailto:bbbraden@asu.edu)

**SUPPLEMENTARY MATERIAL**

**1** **Supplementary Methods**

**1.1** **Data Quality Control (QC) and Participant Inclusion**

**1.1.1 Initial Data Screening and Exclusion**

Prior to any processing, all raw T1-weighted and diffusion-weighted images were visually inspected using MRIcron (https://www.nitrc.org/projects/mricron/) and fsleyes. Participants were excluded at this stage due to: (a) incomplete image acquisition, (b) excessive motion artifacts, (c) blurring, or (d) pronounced signal noise that precluded reliable analysis.

**1.1.2 QC for Diffusion MRI Processing and TBSS**

Following the calculation of diffusion tensor metrics (including FA for DFA and diffusivities for ALPS index), all individual FA maps were again visually inspected. Subjects whose maps exhibited critical artifacts (e.g., severe signal dropout, ghosting, or distortions arising from residual eddy currents or motion) were excluded. Participants for whom the DFA pipeline failed to generate valid output were also excluded from further morphological and network analyses.

**1.1.3 QC for Structural MRI Processing**

The automated outputs from FreeSurfer for cortical and subcortical segmentation were meticulously reviewed before statistical analysis. This involved visual inspection of the white matter and pial surfaces, as well as subcortical segmentations, in multiple planes. Participants with inaccurate segmentation or surface reconstruction, identified by clear deviations from anatomical boundaries, were excluded from all analyses involving morphological data.

**1.1.4 QC for DTI-ALPS Index Calculation**

The quality control protocol for DTI-ALPS analysis included systematic visual inspection of all key diffusion metrics. Specifically, the diffusivity maps (Dxx, Dyy, Dzz), along with intermediate processed images such as the eddy-corrected images and FA maps, were reviewed in both native and standardized template spaces. Any potential processing errors or images of poor quality were identified and excluded. This step aimed to identify and exclude any cases where ROI placement was erroneous due to processing artifacts or where the underlying diffusivity maps were of poor quality.

**1.1.5 Matching of Healthy Controls**

Following the application of this sequential QC protocol, a total of 25 patients were excluded. Specifically, from the initial cohort of 76 bvFTD patients in the FTLDNI database: 10 patients were excluded owing to incomplete image acquisition or poor image quality (e.g., pronounced signal noise); 4 were excluded due to failure in DFA computation; 10 were excluded because of inaccurate cortical surface reconstruction in the temporal and/or frontal lobes; and 1 was excluded due to a failure in the DTI-ALPS processing pipeline. Consequently, 51 bvFTD patients with complete and validated data across all imaging modalities (structural, diffusion, DTI-ALPS) remained for the final integrated analyses. Furthermore, to ensure statistically balanced group comparisons and mitigate potential bias from unequal sample sizes, a demographically matched control group was constructed. Each of the 51 included bvFTD patients was matched with one healthy control from a larger available pool based on key variables: age and sex. This resulted in a final analytic sample of 51 bvFTD patients and 51 demographically matched healthy controls for all primary case-control comparisons. The remaining unmatched controls were not utilized in the main analyses to preserve this balance and analytical rigor.

**1.2 DFA Processing Protocol**

**1.2.1 Implementation of Computational Analysis**

All analyses were conducted using a standardized preprocessing pipeline based on FSL (https://fsl.fmrib.ox.ac.uk/fsl). To ensure full computational reproducibility, the complete software environment was encapsulated and deployed as a Singularity container image (dmritool-ubuntu-20210612). All processing commands were executed within this standardized container environment. The DFA methodology was based on the framework described by Cheng and Basser ^1^. Identical parameter configurations were applied uniformly across all subjects during processing. All intermediate and final output files were preserved in the NIfTI format to facilitate subsequent group-level statistical analysis and visualization.

**1.2.2 Data Standardization and Preprocessing**

First, all diffusion-weighted MRI data underwent standardized preprocessing using FSL, including corrections for eddy currents and head motion, and brain extraction. Then, within a Singularity container environment, the orientation information was stripped from the NIfTI-format images using FSL commands to enforce a standard spatial orientation. Subsequently, the b-value and gradient vector files were converted from a columnar to a row-wise format, generating a unified DWI configuration file for subsequent processing. A whole-brain tissue mask was automatically generated using Brain Extraction Tool (BET) with standard parameters to define the region of interest for all subsequent analyses. The specific commands and key parameters are as follows:

1. **Transposition of b-values and gradient vectors**

*TextFileOperator subject.bval --transpose -o b.txt*

*TextFileOperator subject.bvec --transpose -o grad.txt*

1. **Image orientation standardization**

*fslorient -deleteorient subject.nii.gz*

1. **Generation of DWI file**

*echo b.txt grad.txt subject.nii.gz >> dwi.txt*

1. **Generation of brain tissue mask**

*bet subject.nii.gz mask.nii.gz*

**1.2.3 DTI Analysis**

Following data preprocessing, the diffusion tensor was estimated from the preprocessed diffusion-weighted images, with calculations constrained to voxels within the brain tissue mask. The calculation of diffusion metrics yielded maps for fractional anisotropy (FA) and mean diffusivity (MD). The specific commands and key parameters are as follows:

1. **Tensor estimation**

*DWIToTensor dwi.txt dti.nii.gz --mask mask.nii.gz*

1. **Calculation of diffusion metrics**

*TensorToFeatures dti.nii.gz --fa fa.nii.gz --md md.nii.gz --mask mask.nii.gz*

**1.2.4 DFA Analysis**

First, the principal eigenvector field (the primary diffusion direction) was extracted from the diffusion tensor. To exclude isotropic diffusion regions, an FA threshold of = 0.2 (or 0.3) was applied, thereby filtering out voxels with FA values below this threshold. Subsequently, the primary diffusion directions were transformed into a local coordinate frame representation, upon which the four DFA metrics (splay, bend, twist, and total distortion) were computed. The specific commands and key parameters are as follows:

1. **Extraction of principal eigenvector (FA = 0.2 or 0.3)**

*TensorToFeatures dti.nii.gz --v1 dti_v1.nii.gz --fathreshold 0.2*

1. **Transformation to local coordinate frame**

*PeaksToLocalFrame dti_v1.nii.gz dti_frame.nii.gz --peaktype XYZ*

1. **Calculation of DFA metrics**

*LocalFrameToFeatures dti_frame.nii.gz --splay dti_frame_splay.nii.gz --bend dti_frame_bend.nii.gz --twist dti_frame_twist.nii.gz --distortion dti_frame_distortion.nii.gz*

**1.3 Detailed Description of Network Efficiency Metrics**

- - 1. **Global efficiency (Eg)**

Global efficiency measures the average efficiency of information transfer between all pairs of nodes in a network. It reflects the overall integration capability or global information processing capacity of the network.

For a pair of nodes $i$ and *j* in a network *G*, their efficiency $\epsilon_{ij}$ is defined as the reciprocal of the shortest-path distance $L_{i,j}$ between them. If no path exists between $i$ and *j* (i.e., $L_{i,j}=\infty$), then $\epsilon_{ij}=0$.

$$\begin{aligned} E_{\mathrm{global}}=\frac{1}{N\left( N-1 \right)}\sum_{i\neq j\in G} \epsilon_{ij}=\frac{1}{N\left( N-1 \right)}\sum_{i\neq j\in G} \frac{1}{L_{i,j}} \#\left( 1 \right) \end{aligned}$$

where *N* is the total number of nodes in the network. Its value ranges within [0, 1]. For a fully connected network where the distance between any two nodes is 1, the global efficiency attains its maximum value of 1.

**1.3.2** **Local efficiency (Eloc)**

Local efficiency measures the network’s fault tolerance against local disruptions. Specifically, it computes the global efficiency of the neighborhood subgraph (i.e., the network formed by the direct neighbors of a node, excluding the node itself) for each node and then averages this value across all nodes. It reflects the backup capacity for communication among a node’s neighbors when that node fails.

For a node $i$, the local efficiencyof node $i$ is defined as the global efficiency of subgraph $G_{i}:$

$$\begin{aligned} E_{local}=\frac{1}{N_{G_{i}}\left( N_{G_{i}}-1 \right)}\sum_{j,k\in G_{i}} \frac{1}{L_{j,k}} \#\left( 2 \right) \end{aligned}$$

where$N_{G_{i}}$ is the total number of nodes in the network $G_{i}$.

- - 1. **Shortest Path Length (Lp)**

Shortest path length measures the average shortest-path distance between all pairs of nodes in a network. It reflects the overall ease or cost of global information transfer, with shorter lengths indicating higher global integration efficiency.

For a pair of nodes $i$ and $j$ in a network $G$, their shortest-path distance $d_{ij}$ is defined as the minimum number of edges that must be traversed to connect them. If no path exists between $i$ and $j$ (i.e., the nodes belong to disconnected components), then $d_{ij}=\infty$.

$$\begin{aligned} L_{p}=\frac{1}{N\left( N-1 \right)}\sum_{i\neq j\in G} d_{ij}\#\left( 3 \right) \end{aligned}$$

where $N$ is the total number of nodes in the network. The characteristic path length is defined only for connected graphs or connected components. For a fully connected network where the distance between any two nodes is 1, $L_{p}$ attains its minimum value of 1. Conversely, a longer $L_{p}$ indicates a less efficiently integrated network.

- - 1. **Clustering Coefficient (Cp)**

Clustering coefficient measures the degree to which nodes in a network tend to cluster together, reflecting the network’s local segregation and potential for modular or specialized processing.

For a node $i$ in a network $G$ with $k_{i}$ neighbors, its local clustering coefficient $C_{i}$ quantifies the proportion of connections that exist among its neighbors relative to all possible connections between them. The local clustering coefficient for node $i$ is calculated as:

$$\begin{aligned} C_{i}=\frac{2E_{i}}{k_{i}\left( k_{i}-1 \right)}\#\left( 4 \right) \end{aligned}$$

where $E_{i}$ is the number of edges that actually exist among the $k_{i}$ neighbors of node $i$, and $k_{i}(k_{i}-1)/2$ is the maximum possible number of such edges.

The global clustering coefficient $C_{p}$ is then defined as the average of the local clustering coefficients across all nodes in the network:

$$\begin{aligned} C_{p}=\frac{1}{N}\sum_{i\in G} C_{i}\#\left( 5 \right) \end{aligned}$$

where $N$ is the total number of nodes. Its value ranges within$[0,1]$. A value of 1 indicates a perfectly clustered network where every node’s neighbors form a complete subgraph (clique), while a value of 0 indicates a tree-like structure with no local triangles. Higher $C_{p}$ values indicate greater local interconnectedness and network segregation.

- - 1. **Nodal efficiency**

Nodal efficiency, also referred to as nodal global efficiency centrality, measures the average efficiency of information exchange between a single node and all other nodes in the network. It reflects the importance or accessibility of that node in global network communication. A node with high nodal efficiency indicates that its average shortest‑path length to all other nodes is short, meaning it occupies a central position in the network.

The nodal efficiency $E_{\text{nodal}}(i)$ is defined as the average efficiency from node $i$ to all other nodes in the network:

$$\begin{aligned} E_{\text{nodal}}\left( i \right)=\frac{1}{N-1}\sum_{j\in G} \frac{1}{L_{i,j}}\#\left( 6 \right) \end{aligned}$$

**2. Supplementary Figures**


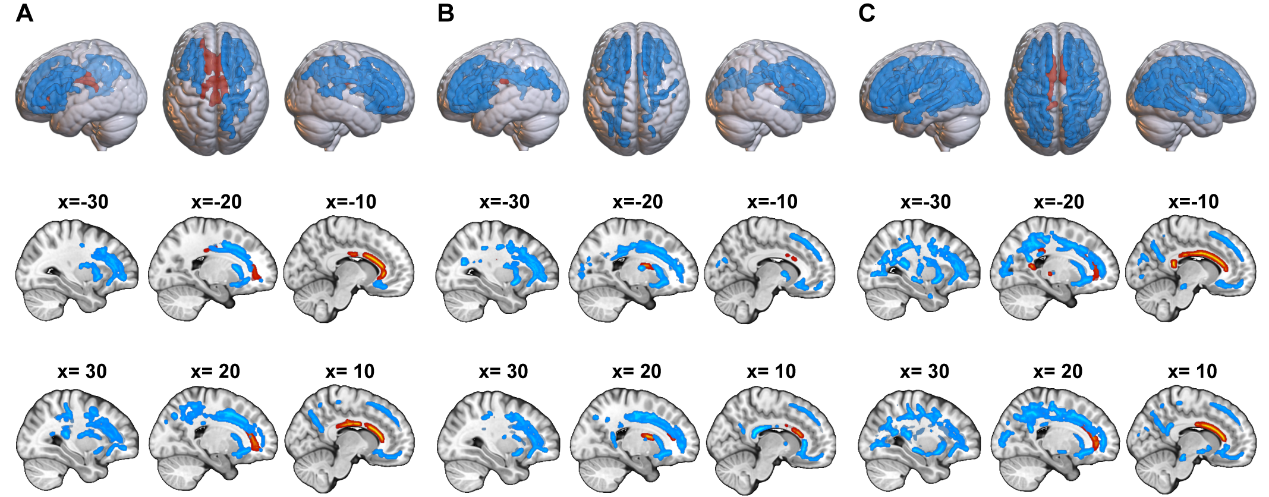


**Supplementary Figure 1.** Brain regions showing significant differences in **(A)** splay, **(B)** bend and **(C)** twist between patients with bvFTD (N=51) and HCs (N=51). Between-group comparisons were performed using a nonparametric permutation test (5000 repetitions), adjusting for age and gender. Cool colors indicate regions where patients with bvFTD had significantly lower values in the corresponding metric compared to HCs, while warm colors indicate regions with significantly higher values in patients with bvFTD than in HCs. The results were obtained with a threshold of TFCE-corrected p<0.05.

**
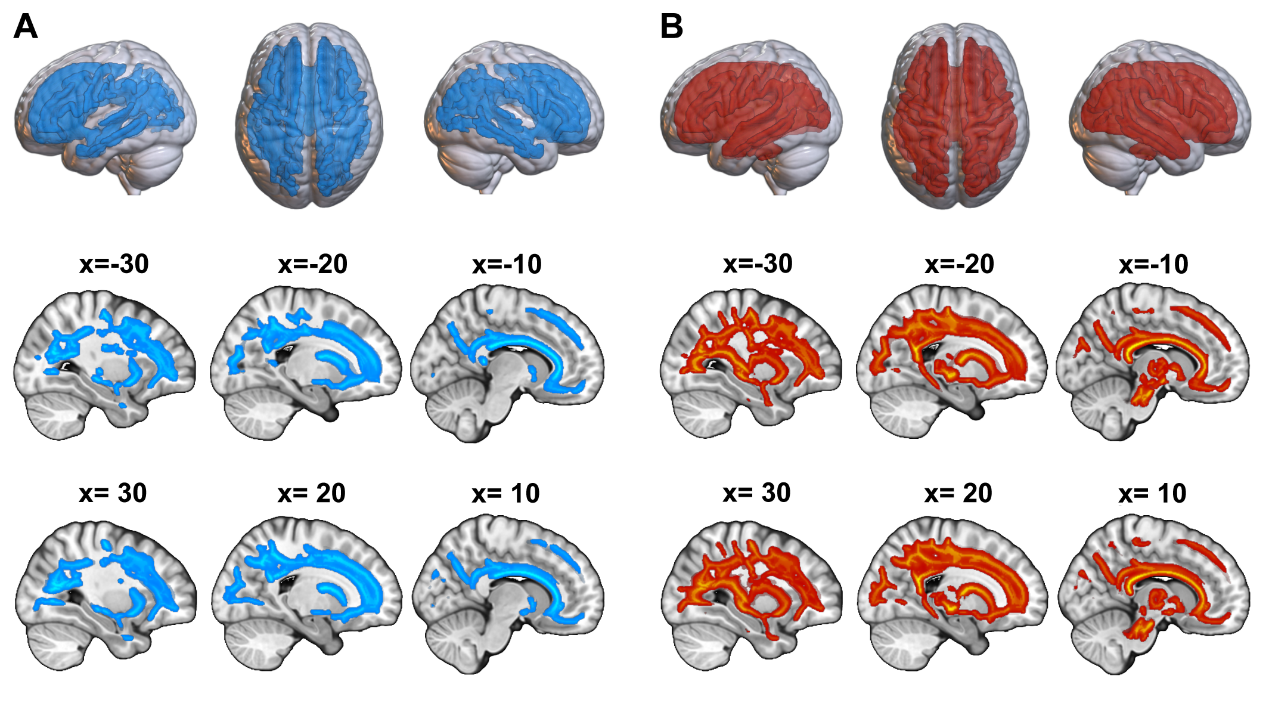
**

**Supplementary Figure 2.** Brain regions showing significant differences in **(A)** FA and **(B)** MD between patients with bvFTD (N=51) and HCs (N=51). Between-group comparisons were performed using a nonparametric permutation test (5000 repetitions), adjusting for age and gender. Cool colors indicate regions where patients with bvFTD had significantly lower values in the corresponding metric compared to HCs, while warm colors indicate regions with significantly higher values in patients with bvFTD than in HCs. The results were obtained with a threshold of TFCE-corrected p<0.05.

**
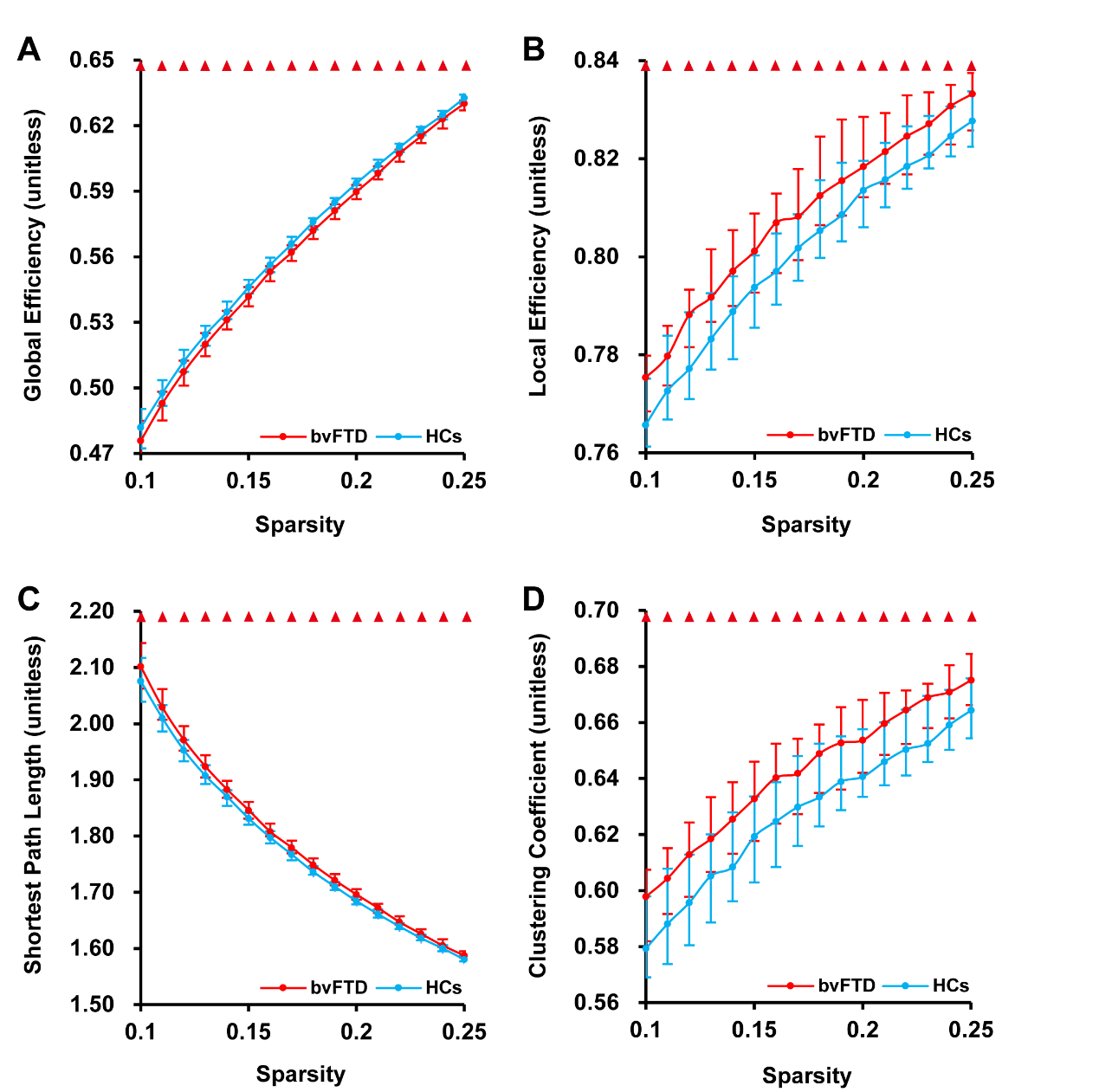
**

**Supplementary Figure 3.** Differences in global network parameters between patients with bvFTD (N=51) and HCs (N=51). **(A)** global efficiency, **(B)** local efficiency, **(C)** shortest path length and **(D)** clustering coefficient in patients with bvFTD were compared with those in HCs at different network sparsity levels using Mann-Whitney tests. All data are presented as median (25% fraction, 75% fraction). Black upward triangles (▲) represent significant differences (p < 0.05, two-tailed).

**3. Supplementary Tables**

**Supplementary Table 1.** Differences in imaging metrics between patients with bvFTD and HCs.

| **Variables** | **bvFTD** | **HCs** | ***p*-value** |
| --- | --- | --- | --- |
| WM | 436275.00(380923.25,461317.81) | 460102.00(380923.25,508721.40) | 0.0026 |
| GM | 569798.00(513192.00,610145.00) | 616718.00(586986.80,663626.50) | <0.0001 |
| CP | 1910.00(1589.48,2127.35) | 1394.00(1156.90,1641.00) | <0.0001 |
| LV | 45630.00(39611.93,57150.55) | 23269.00(18450.30,34485.15) | <0.0001 |
| Sigma | 0.3531(0.3460,0.3643) | 0.3481(0.3402,0.3544) | 0.0232 |
| Gamma | 0.4023(0.3823,0.4168) | 0.3890(0.3807,0.3976) | 0.0153 |
| Lamdba | 0.1677(0.1658,0.1695) | 0.1667(0.1652,0.1674) | 0.0065 |
| Eg | 0.0755(0.0749,0.0764) | 0.0764(0.0759,0.0768) | 0.0010 |
| Eloc | 0.1178(0.1167,0.1188) | 0.1163(0.1156,0.1176) | 0.0014 |
| Cp | 0.0913(0.0898,0.0935) | 0.0893(0.0875,0.0916) | 0.0011 |
| Lp | 0.3020(0.2984,0.3045) | 0.2984(0.2965,0.3006) | 0.0015 |
| ALPS | 1.221(1.159322,1.297866) | 1.369(1.254598,1.440247) | <0.0001 |

**Note:** Imaging metrics were compared between the two groups using a Mann-Whitney Test. Morphological features (WM, GM, CP, and LV) were quantified in mm³. All data represent median (25% fraction, 75% fraction).

**Abbreviations:** WM, Cerebral White Matter; GM, Total Grey Matter; CP, Choroid Plexus; LV, Lateral Ventricle; Sigma, Small-worldness; Gamma, Normalized Clustering Coefficient; Lambda, Normalized Characteristic Path Length; Eg, Global Efficiency; Eloc, Local Efficiency; Cp, Clustering Coefficient; Lp, Shortest Path Length.

**Supplementary Table 2.** Results of Mann-Whitney tests comparing global network parameters between bvFTD patients (N=51) and HCs (N=51) across network sparsity levels (0.1-0.25, step size = 0.01).

| **Network Sparsity Level** | **U Statistics** | **Z-value** | **P-values** |
| --- | --- | --- | --- |
| **global efficiency** | | | |
| 0.10 | 1715 | -2.0868 | 0.0369 |
| 0.11 | 1641 | -2.6750 | 0.0075 |
| 0.12 | 1663.5 | -2.4962 | 0.0126 |
| 0.13 | 1657 | -2.5478 | 0.0108 |
| 0.14 | 1553 | -3.3746 | 0.0007 |
| 0.15 | 1519.5 | -3.6409 | 0.0003 |
| 0.16 | 1526.5 | -3.5853 | 0.0003 |
| 0.17 | 1478.5 | -3.9669 | 0.0001 |
| 0.18 | 1455 | -4.1537 | <0.0001 |
| 0.19 | 1486.5 | -3.9033 | 0.0001 |
| 0.20 | 1464 | -4.0822 | 0.0000 |
| 0.21 | 1476.5 | -3.9828 | 0.0001 |
| 0.22 | 1503.5 | -3.7682 | 0.0002 |
| 0.23 | 1461.5 | -4.1021 | <0.0001 |
| 0.24 | 1505 | -3.7562 | 0.0002 |
| 0.25 | 1487.5 | -3.8954 | 0.0001 |
| **local efficiency** | | | |
| 0.10 | 2241 | 2.0868 | 0.0369 |
| 0.11 | 2315 | 2.6750 | 0.0075 |
| 0.12 | 2292.5 | 2.4962 | 0.0126 |
| 0.13 | 2299 | 2.5478 | 0.0108 |
| 0.14 | 2403 | 3.3746 | 0.0007 |
| 0.15 | 2436.5 | 3.6409 | 0.0003 |
| 0.16 | 2429.5 | 3.5853 | 0.0003 |
| 0.17 | 2477.5 | 3.9669 | 0.0001 |
| 0.18 | 2501 | 4.1537 | <0.0001 |
| 0.19 | 2469.5 | 3.9033 | 0.0001 |
| 0.20 | 2492 | 4.0822 | <0.0001 |
| 0.21 | 2479.5 | 3.9828 | 0.0001 |
| 0.22 | 2452.5 | 3.7682 | 0.0002 |
| 0.23 | 2494.5 | 4.1021 | <0.0001 |
| 0.24 | 2451 | 3.7562 | 0.0002 |
| 0.25 | 2468.5 | 3.8954 | 0.0001 |
| **shortest path length** | | | |
| 0.10 | 2241 | 2.0868 | 0.0369 |
| 0.11 | 2315 | 2.6750 | 0.0075 |
| 0.12 | 2292.5 | 2.4962 | 0.0126 |
| 0.13 | 2299 | 2.5478 | 0.0108 |
| 0.14 | 2403 | 3.3746 | 0.0007 |
| 0.15 | 2436.5 | 3.6409 | 0.0003 |
| 0.16 | 2429.5 | 3.5853 | 0.0003 |
| 0.17 | 2477.5 | 3.9669 | 0.0001 |
| 0.18 | 2501 | 4.1537 | <0.0001 |
| 0.19 | 2469.5 | 3.9033 | 0.0001 |
| 0.20 | 2492 | 4.0822 | <0.0001 |
| 0.21 | 2479.5 | 3.9828 | 0.0001 |
| 0.22 | 2452.5 | 3.7682 | 0.0002 |
| 0.23 | 2494.5 | 4.1021 | 0.0000 |
| 0.24 | 2451 | 3.7562 | 0.0002 |
| 0.25 | 2468.5 | 3.8954 | 0.0001 |
| **Clustering Coefficient** | | | |
| 0.10 | 2340 | 2.8738 | 0.0041 |
| 0.11 | 2304 | 2.5876 | 0.0097 |
| 0.12 | 2369 | 3.1043 | 0.0019 |
| 0.13 | 2374 | 3.1440 | 0.0017 |
| 0.14 | 2379 | 3.1838 | 0.0015 |
| 0.15 | 2325 | 2.7545 | 0.0059 |
| 0.16 | 2349 | 2.9453 | 0.0032 |
| 0.17 | 2334 | 2.8261 | 0.0047 |
| 0.18 | 2356 | 3.0010 | 0.0027 |
| 0.19 | 2317 | 2.6909 | 0.0071 |
| 0.20 | 2345 | 2.9135 | 0.0036 |
| 0.21 | 2353 | 2.9771 | 0.0029 |
| 0.22 | 2377 | 3.1679 | 0.0015 |
| 0.23 | 2369 | 3.1043 | 0.0019 |
| 0.24 | 2353 | 2.9771 | 0.0029 |
| 0.25 | 2341 | 2.8817 | 0.0040 |

**Note:** U: Mann-Whitney statistic; Z: standardized test statistic; P: two-tailed p-value.

**Supplementary Table 3.**Associations between imaging and clinical data in patients with bvFTD

| **Variables** | **CDR** | **MMSE** |
| --- | --- | --- |
| Splay Decrease | r =-0.6629, p < 0.0001 | r = 0.3629, p = 0.0096 |
| Bend Decrease | r =-0.6788, p < 0.0001 | r = 0.3644, p = 0.0089 |
| Twist Decrease | r =-0.6844, p < 0.0001 | r = 0.3642, p = 0.0093 |
| Distortion Decrease | r =-0.6782, p < 0.0001 | r = 0.3474, p = 0.0134 |
| Splay Increase | r =-0.6789, p < 0.0001 | r = 0.3370, p = 0.0167 |
| Bend Increase | r =-0.6163, p < 0.0001 | r = 0.3886, p = 0.0053 |
| Twist Increase | r =-0.6861, p < 0.0001 | r = 0.3647, p = 0.0092 |
| Distortion Increase | r =-0.6329, p < 0.0001 | r = 0.3571, p = 0.0109 |
| Cerebral White Matter | r =-0.4552, p = 0.0009 | ns |
| Total Grey Matter | r =-0.2958, p = 0.0370 | ns |
| Choroid Plexus | ns | r =-0.3394, p = 0.0159 |
| Lateral Ventricle | r = 0.3393, p = 0.0159 | r =-0.3653, p = 0.0091 |

**Note:** The results were assessed using Pearson’s correlation coefficient. The ‘ns’ indicates that the result of the correlation analysis is not statistically significant.

**Supplementary Table 4.** Associations between DFA metrics and other imaging parameters in patients with bvFTD.

| **Variables** | **EG** | **LP** | **WM** | **GM** | **CP** | **LV** |
| --- | --- | --- | --- | --- | --- | --- |
| Splay Decrease | r = 0.3396  p = 0.0259 | r =-0.3385  p = 0.0264 | r = 0.4854  p = 0.0004 | ns | ns | ns |
| Bend Decrease | r = 0.3339  p = 0.0287 | r =-0.3295  p = 0.0310 | r = 0.5256  p < 0.0001 | ns | ns | ns |
| Twist Decrease | r = 0.3437  p = 0.0240 | r =-0.3378  p = 0.0267 | r = 0.4693  p = 0.0006 | ns | ns | ns |
| Distortion Decrease | r = 0.3398  p = 0.0258 | r =-0.3342  p = 0.0285 | r = 0.4668  p = 0.0006 | ns | ns | ns |
| Splay Increase | ns | ns | r = 0.6764  p < 0.0001 | r = 0.4627  p = 0.0007 | r =-0.3111  p = 0.0279 | r =-0.3505  p = 0.0125 |
| Bend Increase | ns | ns | r = 0.6770  p < 0.0001 | r = 0.4745  p = 0.0005 | r =-0.3090  p = 0.0290 | r =-0.2852  p = 0.0447 |
| Twist Increase | ns | ns | r = 0.6800  p < 0.0001 | r = 0.4669  p = 0.0005 | r =-0.3460  p = 0.0139 | r =-0.3751  p = 0.0073 |
| Distortion Increase | ns | ns | r = 0.6855  p < 0.0001 | r = 0.4861  p = 0.0003 | r =-0.3323  p = 0.0184 | r =-0.3566  p = 0.0110 |

**Note:** The results were assessed using Pearson’s correlation coefficient. The ‘ns’ indicates that the result of the correlation analysis is not statistically significant.

**Abbreviations:** Eg, Global Efficiency; Lp, Shortest Path Length; WM, Cerebral White Matter; GM, Total Grey Matter; CP, Choroid Plexus; LV, Lateral Ventricle.

**Supplementary reference**

1. Cheng J, Basser PJ. Director Field Analysis (DFA): Exploring Local White Matter Geometric Structure in Diffusion MRI. *Med Image Anal*. Jan 2018;43:112-128. doi:10.1016/j.media.2017.10.003
